# Hypoxia-pyroptosis-associated long noncoding RNAs as prognostic biomarkers in lung cancer: a systematic review and meta-analysis

**DOI:** 10.36416/1806-3756/e20250525

**Published:** 2026-09-14

**Authors:** Chaonan Ma, Misbah Ullah Khan

**Affiliations:** 1. Department of Respiratory and Critical Care Medicine, Huaihe Hospital of Henan University,Kaifeng, China.; 2. Centre for Nanosciences, University of Okara, Okara, Pakistan.

**Keywords:** Lung neoplasms, RNA, long noncoding, Hypoxia, Pyroptosis

## Abstract

**Objective:**

Hypoxia-pyroptosis-associated long noncoding RNAs (HP-lncRNAs) may contribute to cancer progression and treatment resistance, but their prognostic and mechanistic roles in lung cancer remain unclear. We conducted a systematic review and meta-analysis to evaluate their prognostic value and regulatory networks in lung cancer.

**Methods:**

Following Preferred Reporting Items for Systematic Reviews and Meta-Analyses guidelines, we systematically searched five scientific databases up to November 20, 2025, for studies evaluating HP-lncRNA expression and survival outcomes. Hazard ratios (HRs) were pooled by using random-effects models, with heterogeneity being assessed by I^2^. Subgroup analyses examined histology, tumor stage, and study design. Mechanistic evidence and protein-interaction networks were also synthesized.

**Results:**

A total of 27 studies were included, with 14 providing quantitative data for meta-analysis. High expression of HP-lncRNA signatures was significantly associated with poorer overall survival (pooled adjusted HR = 1.50; 95% CI, 1.23-1.84), with a pronounced effect in lung adenocarcinoma (adjusted HR = 1.64; 95% CI, 1.34-1.99). Subgroup analyses supported a consistent adverse association across cohort designs and showed that the association remained evident in the advanced-stage subgroup (adjusted HR = 1.60; 95% CI, 1.44-1.77), although clear stage-specific effect modification was not demonstrated. Mechanistic evidence linked some lncRNAs to hypoxia-related regulation and others to pyroptosis-related signaling. Although direct evidence of a single lncRNA connecting both pathways is limited, findings support possible hypoxia-pyroptosis crosstalk. HIF-1a and caspase-1 emerged as central hub genes.

**Conclusions:**

Our findings suggest that HP-lncRNA signatures may have prognostic relevance and therapeutic potential in lung cancer; however, the marked between-study heterogeneity indicates that further standardization and external validation are required before clinical application.

## INTRODUCTION

Lung cancer is the leading cause of global cancer mortality, with over 2.48 million new cases and 1.81 million deaths estimated in 2022.^1^ The overall five-year survival rate for patients with lung cancer remains low, which is largely due to factors including significant tumor heterogeneity, rapid progression, and the development of therapeutic resistance.^2^

A hallmark of the solid tumor microenvironment (TME) is hypoxia, a state of reduced oxygen tension (typically < 10 mmHg) resulting from imbalanced angiogenesis and rapid tumor growth.^3^ The hypoxic condition in the TME is linked to increased tumor invasion, metastatic ability, immune evasion, and resistance to chemotherapy and radiation therapy.^4^ The cellular response to hypoxia is primarily mediated by hypoxia-inducible factors (HIFs). These transcription factors coordinate gene networks that promote angiogenesis and epithelial-mesenchymal transition, thereby facilitating metastasis.^5^ Recently, research has focused on how hypoxia modulates inflammatory programmed cell death, specifically pyroptosis.^6^ Distinct from apoptosis or necroptosis, pyroptosis is characterized by gasdermin-mediated pore formation in the plasma membrane and the release of inflammatory cytokines such as IL-1β and IL-18.^7^ Emerging evidence suggests that hypoxic signaling suppresses this pathway to ensure tumor survival; specifically, HIFs can inhibit the expression and activation of gasdermin family members, including gasdermin D (GSDMD), gasdermin E (GSDME), and gasdermin C (GSDMC).^8^ ^,^ ^9^

Long noncoding RNAs (lncRNAs) have emerged as essential regulators within this hypoxia-pyroptosis axis. They influence cancer gene expression through diverse mechanisms, including chromatin remodeling, transcriptional regulation, and post-transcriptional silencing.^10^ In lung cancer, hypoxia-inducible lncRNAs such as *MALAT1* and *NEAT1* have been shown to epigenetically silence the NLR family pyrin domain containing 3 (NLRP3)/GSDMD pathway.^11^ ^,^ ^12^ These lncRNAs represent a promising therapeutic target to re-engage pyroptotic cell death and improve immunotherapy efficacy.^12^ ^,^ ^13^ In addition, recent systematic reviews of blood-based biomarkers have suggested that DNA methylation is associated with increased lung cancer risk, whereas mitochondrial DNA copy number remains inconclusive, underscoring the potential value of multimarker approaches that combine complementary molecular signals.^14^ ^,^ ^15^

Despite growing interest, current literature often examines hypoxia signaling, lncRNA biology, and pyroptosis separately, lacking an integrated synthesis. To address this gap, we conducted a systematic review and meta-analysis to comprehensively evaluate the prognostic significance of hypoxia-pyroptosis-associated lncRNAs (HP-lncRNAs) in lung cancer. By synthesizing clinical and experimental evidence, we sought to establish a unified mechanistic framework; assess the diagnostic and therapeutic potential of HP-lncRNAs; and provide a translational foundation for targeting this axis in lung cancer management. 

## METHODS

### Search strategy and data sources

The protocol for the present systematic review was registered with the International Prospective Register of Systematic Reviews (PROSPERO ID: CRD420251156050). In accordance with Preferred Reporting Items for Systematic Reviews and Meta-Analyses (PRISMA) guidelines, we systematically searched scientific databases including EMBASE, PubMed, Scopus, Web of Science, and Google Scholar from inception through November 20, 2025, to identify studies evaluating the role of HP-lncRNAs in lung cancer. Key search terms included the following: “lung cancer,” “lung carcinoma,” “NSCLC,” “lncRNA,” “long noncoding RNA,” “hypoxia,” “HIF-1α,” “hypoxic microenvironment,” “pyroptosis,” “inflammasome,” “Gasdermin,” “NLRP3,” and “prognosis.” The reference lists of all included studies and relevant review articles were manually screened to identify additional eligible publications. Non-English language publications were excluded. 

### Study selection criteria

Studies were eligible for inclusion if they met the following criteria: (1) being original research articles (prospective or retrospective cohorts) involving lung cancer patients; (2) evaluating lncRNA expression using validated high-throughput (e.g., RNA sequencing) or molecular (e.g., quantitative RT-PCR) platforms; (3) providing survival outcome data, including hazard ratios (HRs) with 95% CIs, or other data to calculate HRs; (4) providing either experimental (e.g., hypoxia-inducible factor 1-alpha [HIF-1α] assessment, hypoxia cell models, and hypoxia-related gene signatures) or computational (e.g., correlation with hypoxia gene sets) evidence of hypoxia involvement; and (5) providing experimental or computational evidence linking to pyroptosis (e.g., caspase-1 activation, GSDMD cleavage, IL-1β/IL-18 quantification, and NLRP3 inflammasome activity). In vitro and in vivo mechanistic studies relevant to hypoxia-related lncRNA biology, pyroptosis-related lncRNA biology, or their potential crosstalk in lung cancer were included in the qualitative synthesis. For this narrative component, studies were eligible if they provided experimental evidence for at least one of the two biological domains (hypoxia or pyroptosis) in relation to a lung cancer-relevant lncRNA. Studies that assessed both processes within the same experimental system were considered direct integrative evidence, whereas studies addressing only one component were retained as indirect mechanistic evidence. Exclusion criteria included the following: editorials, reviews, case reports, conference abstracts, studies with insufficient methodological detail, and studies not providing extractable prognostic data. 

### Data extraction and quality assessment

Data extraction was performed independently by two reviewers using a standardized, pre-piloted form. Any discrepancies were resolved through re-review of the original studies by both authors and discussion until consensus was reached. The following variables were extracted from each eligible study: first author; publication year; country; study design; sample size; patient demographic characteristics; histological subtype (lung adenocarcinoma [LUAD], lung squamous cell carcinoma [LUSC], or mixed); tumor stage distribution; specific lncRNAs investigated; method of lncRNA expression quantification; type of hypoxia and pyroptosis validation performed; and survival data. For prognostic studies, both crude and adjusted HRs for overall survival (OS) with corresponding 95% CIs, the number of events, and covariates included in multivariable models were recorded. 

The methodological quality of the included cohort studies was assessed with the Newcastle-Ottawa Scale (NOS). The NOS evaluates studies across three domains: (1) selection of the study groups; (2) comparability of the groups; and (3) assessment of the outcome. A scoring system was applied, whereby studies were classified as having a low risk of bias (scores of 7-9), a moderate risk of bias (scores of 4-6), or a high risk of bias (scores below 4). 

### Statistical analysis

All statistical analyses were performed with Stata, version 18 (StataCorp LLC, College Station, TX, USA), following the guidelines outlined in the Cochrane Handbook for Systematic Reviews of Interventions for methodological rigor and reproducibility.^16^ The primary outcome was OS, as measured by HRs with 95% CIs. Study-specific crude and adjusted HRs were pooled separately. Between-study heterogeneity was assessed with Cochran’s Q test (which was considered significant at p < 0.10) and quantified with the I^2^ statistic, which was interpreted as follows: 25%, low; 50%, moderate; and 75%, high.^17^ Given the anticipated clinical and methodological diversity across studies, the DerSimonian-Laird random-effects model was specified a priori for all meta-analyses to provide a more conservative estimate that accounted for heterogeneity.^18^ Pooled estimates are presented with their 95% CIs and two-sided p values, with statistical significance set at p < 0.05. 

To explore sources of heterogeneity and assess the consistency of findings, pre-specified subgroup analyses were performed on the basis of the following: (1) histological subtype (LUAD vs. LUSC); (2) analytical cohort design (training, validation, or combined cohorts); and (3) tumor stage (I-II vs. III-IV), where data were available. Between-subgroup differences were evaluated by using meta-regression. Age and sex were not analyzed as effect modifiers because the included studies reported these variables inconsistently across multivariable models, making pooled interaction-level interpretation inappropriate. The robustness of the primary pooled estimates was examined through a leave-one-out sensitivity analysis, wherein each study was sequentially removed to evaluate its influence on the overall result. The potential for small-study effects and publication bias was assessed through visual inspection of funnel plots for asymmetry and formally tested by using Egger’s weighted regression test.^19^ If significant bias was indicated (Egger’s test p < 0.05), the nonparametric trim-and-fill method was applied to impute theoretically missing studies and compute an adjusted pooled estimate. The reporting of these statistical methods follows established guidelines for meta-analyses to ensure completeness and reproducibility.

### Systematic review

In addition to the quantitative random-effects meta-analysis, we performed a qualitative systematic review to examine mechanistic evidence related to hypoxia-responsive lncRNAs, pyroptosis-regulating lncRNAs, and their possible interplay in lung cancer. This qualitative synthesis was designed as a narrative mapping exercise rather than a formal demonstration that every included study established direct hypoxia-pyroptosis coupling for the same lncRNA within a single model. We extracted hypoxia-related findings from studies using approaches such as hypoxia-mimetic models, low-oxygen exposure, HIF-1α assessment, chromatin immunoprecipitation quantitative PCR, and luciferase reporter assays. We also extracted pyroptosis-related findings from studies reporting inflammasome-associated or gasdermin-mediated signaling, including NLRP3 regulation, caspase activation, GSDMD/GSDME-related events, and IL-1β/IL-18 maturation. Studies that assessed both processes within the same experimental system were considered direct integrative evidence, whereas studies examining only one component were considered indirect evidence relevant to the proposed crosstalk model. This approach allowed us to interpret the pooled clinical associations within a mechanistic framework while maintaining a clear distinction between direct and indirect evidence. 

### Integration and functional classification of lncRNAs

The systematic review of published studies allowed us to predict the relationship between hypoxia-pyroptosis-related genes and lncRNAs in lung cancer. Data were extracted from primary studies, supplementary materials, and reported correlation or network analyses. This included lncRNA identifiers, tumor-versus-normal expression patterns, functional roles within prognostic signatures, and associated hypoxia- and pyroptosis-related protein-coding genes. To eliminate repetition, all of the data were restructured so that each lncRNA was represented by a single consolidated item that included supporting studies (authors and years), as well as harmonized expression and functional annotations across studies. Lists of associated genes were combined and deduplicated. 

### Identification of hypoxia- and pyroptosis-related proteins and protein connection analysis

The lncRNAs related to hypoxia and pyroptosis were taken as a whole, and the proteins related to them were extracted respectively in a very comprehensive manner from the primary articles, supplemental data, or associations or network analyses that were directly or strongly reported. Only those proteins that had direct or strong associations were kept, and these included classical hypoxia regulators (e.g., HIF-1α, endothelial PAS domain-containing protein 1, carbonic anhydrase IX, and VEGF-A) and essential pyroptosis factors (e.g. members of the NLRP family, caspase-1/4/5, GSDMD, GSDME, IL-1β, and IL-18). The association between proteins was noted for each study and for each lncRNA to keep the biological context intact. The functional connectivity was analyzed by mapping the extracted proteins onto previously created protein-protein interaction networks (e.g., Search Tool for the Retrieval of Interacting Genes/Proteins-STRING), and then the analysis was restricted to the high-confidence interactions. The networks produced in this way were explored to pinpoint the common hubs and crosstalk modules that connect the hypoxic signaling with the inflammation-dependent pyroptotic pathways. 

### Gene Ontology enrichment analysis

Gene Ontology enrichment analysis was carried out on gene sets associated with lncRNA through the validation platform (STRING). The support given to the various categories, namely, biological processes, cellular functions, and molecular functions, was viewed as statistically significant if the false discovery rate was < 0.05. Further, the results were combined with protein interaction analyses in order to give clarity on the molecular convergence between the processes of hypoxia and pyroptosis in lung adenocarcinoma. 

## RESULTS

### Study selection and characteristics

Our literature searches initially obtained 485 records. After initial screening, 312 records failed to fulfill the inclusion criteria. Eligibility was evaluated by reading the full texts of the remaining 45 articles. Of those, 18 were excluded because of the following: being review articles and book chapters (n = 10); not being quantitative studies providing survival data (HRs and 95% CIs; n = 2); being single nucleotide polymorphism association studies (n = 2); and being completely experimental studies that did not include patient prognostic data (n = 4). Finally, a total of 27 articles (18 clinical studies^20^ ^-^ ^37^ and nine experimental studies^38^ ^-^ ^46^) met the inclusion criteria for our systematic review. Of the 18 clinical studies, 14^20^ ^-^ ^25^ ^,^ ^27^ ^-^ ^33^ ^,^ ^37^ were included in the quantitative meta-analysis. As noted above, the nine eligible experimental studies were included in the qualitative mechanistic synthesis. A PRISMA flowchart (Figure 1) provides the details of the study selection process. 

**Figure 1:**
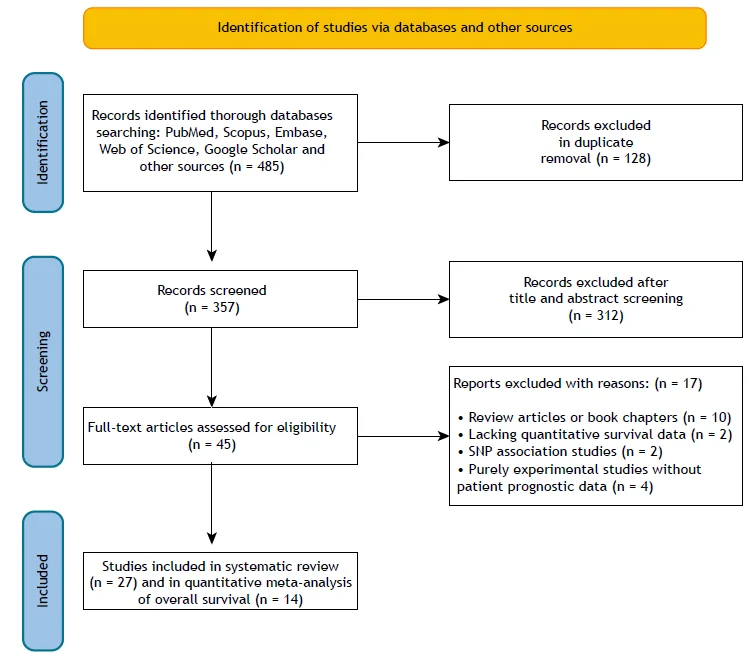
A detailed Preferred Reporting Items for Systematic Reviews and Meta-Analyses (PRISMA) flow diagram illustrating the identification, screening, eligibility assessment, and final inclusion of the 27 studies. SNP: single nucleotide polymorphism.

The 18 included retrospective studies, published between 2021 and 2025, represent a diverse collection of research, including large-scale bioinformatic analyses of public datasets (e.g., The Cancer Genome Atlas and the Gene Expression Omnibus) and retrospective clinical cohort studies with experimental validation. All of the clinical studies (n = 18) were conducted in China. The primary histological subtype investigated was LUAD, followed by LUSC and non-small cell lung cancer (NSCLC). Evidence linking these lncRNAs to hypoxia varied from direct experimental validation (e.g., *HIF1A* chromatin immunoprecipitation) to indirect bioinformatic inference (e.g., gene set enrichment analysis). Similarly, the connection to pyroptosis ranged from direct measurement of key effector proteins (e.g., NLRP3, cleaved caspase-1, and GSDMD) to computational correlation with pyroptosis-related gene sets. On the basis of NOS scores, all 14 studies included in meta-analysis were categorized as high-quality studies (a NOS score ≥ 7). A detailed summary of all included clinical and experimental studies is provided in Chart 1 and Chart 2. 

**Chart 1:** Characteristics of the retrospective studies included in the systematic review and meta-analysis.

Study*	Study Design	Sample Size	Histological Subtype	No. of lncRNAs	Expression Method	Clinical Outcomes	Key Findings and Discussion	NOS Score (Selection/Comparability/Outcome)
Shao et al.^20^sup>	Retrospective, TCGA/GEO-inferred	504 (252 training and 252 validation)	LUAD	7	RNA-seq	Prognostic signature; risk groups with OS differences; immune correlations.	Hypoxia-related lncRNA signature predicts prognosis and TME in LUAD; potential for immune therapy implications.	8 (4/2/2)
Song et al.^21^	Retrospective, TCGA-based	504 (252 training and 252 validation)	LUAD	5	RNA-seq	Risk score: high-risk shorter OS; independent predictor (p < 0.001); nomogram; AUC = 0.757 (1-year).	5-PRlncRNA signature for LUAD prognosis; linked to hypoxia via GSEA; in vitro pyroptosis modulation; potential for personalized therapy.	9 (4/2/3)
Liu et al.^22^	Retrospective with prospective validation	545 (500 TCGA-inferred and 45 validation)	LUAD	3	RNA-seq; quantitative RT-PCR	3-lncRNA risk score: low-risk better OS; independent predictor; nomogram; AUC = 0.775 (1-year); improved immune response in low-risk.	3-PRlncRNA signature validated; low-risk better OS and immunotherapy; role in TME and PD-1/CTLA-4 blockade.	9 (4/2/3)
Peng et al.^23^	Retrospective, TCGA-based	490 (245 training and 245 validation)	LUAD	6	RNA-seq	6-lncRNA signature: low-risk better OS; AUC = 0.742 (1-yr), 0.762 (3-year), 0.749 (5-year); independent factor.	6-lncRNA multi-PCD signature; low-risk favorable TME/immunotherapy; PCD interactions and drug sensitivity clusters.	8 (4/2/2)
Huang et al.^24^	Retrospective, TCGA-based	1,250 (501 training and 749 validation)	LUAD	11	RNA-seq	11-lncRNA signature: high-risk worse OS; immune score differences; HR > 1.	11-PRlncRNA signature predicts OS/immunotherapy; role in TME and checkpoints.	8 (4/2/2)
Li et al.^25^	Retrospective, TCGA-based	499 (TCGA-inferred)	LUAD	7	RNA-seq	Risk model: low-risk better OS; nomogram.	7-hypoxia lncRNA signature for prognosis; links to immune infiltrates and targets.	8 (4/2/2)
Zhou et al.^26^	Retrospective, TCGA-based	495 (248 training and 247 validation)	LUSC	5	RNA-seq	5-lncRNA signature: high-risk worse OS (p < 0.001); high AUC; independent prognostic.	PR signature for LUSC prognosis; links to TME and immunotherapy.	9 (4/2/3)
Wu et al.^27^	Retrospective (inferred)	490	LUAD	6	RNA-seq	Prognostic model: risk groups OS differences.	New PRlncRNA model for LUAD; validation and prognosis emphasis.	7 (3/2/2)
Zhang et al.^28^	Retrospective, TCGA/GEO-based	475 (239 training and 236 validation)	LUSC	11	RNA-seq	11-lncRNA signature: risk groups OS differences; high ROC; nomogram.	PRlncRNA signature for LUSC; links to TME and drug targets.	8 (4/2/2)
Ma et al.^29^	Retrospective, TCGA/GEO-based	1,054 (500 training and 554 validation)	LUAD	8	RNA-seq	8-lncRNA signature (e.g., TSPOAP1-AS1): low-risk better OS; nomogram; HR > 1 for high-risk.	8-cuproptosis lncRNA signature; links to cell death forms including pyroptosis; therapeutic targets.	9 (4/2/3)
Liu et al.^30^	Retrospective, TCGA-based with in vitro analysis	504	LUAD	8	RNA-seq; quantitative PCR (in vitro)	8-lncRNA signature: low-risk better OS; *GSEC* oncogenic in vitro.	Novel IH-related signature; ceRNA axis validated; immune/therapy implications.	9 (4/2/3)
Wang et al.^31^	Retrospective, TCGA-based	468 (236 training and 232 validation)	LUAD	11	RNA-seq	11-lncRNA signature: AUC = 0.778 (1-year); low-risk better OS; HR > 1.	PRlncRNA signature predicts OS, TME, drug sensitivity; prognosis/therapy focus.	8 (4/2/2)
Zhan et al.^32^	Retrospective, TCGA-based	473 (237 training and 236 validation)	LUSC	7	RNA-seq; RT-quantitative PCR	Risk model: low-risk better survival; HR = 1.273 (95% CI, 1.185-1.367; p < 0.001); nomogram; high-risk reduced chemotherapy/immunotherapy response.	7-PRlncRNA signature for LUSC; validated prognosis/TME; MIR193BHG in vitro; clinical application potential.	9 (4/2/3)
Bai et al.^33^	Retrospective, TCGA/GTEx-based	1,170 (498 training and 672 validation)	LUAD	3	RNA-seq	3-pRL model: low-risk better OS; AUC = 0.778 (1-year); nomogram; TMB/TIDE differences.	3-pRL prognostic model; validated by KM/Cox; immune/therapeutic insights.	8 (4/2/2)
Ba et al.^34^	Retrospective, multi-dataset (TCGA + others)	921 (506 training, 181 validation, and 234 validation)	LUAD	11	RNA-seq	Pyroptosis-derived lncRNA signature (random forest optimal); robust OS prediction in cohorts.	Novel pyroptosis-derived lncRNA signature with strong predictive capabilities; insight into LUAD prognosis.	9 (4/2/3)
Guo et al.^35^	Retrospective, TCGA-based	447	LUAD	10	RNA-seq	Prognosis worse with high hypoxia/stemness; ceRNA axis regulates progression.	ceRNA network: AC079160.1-miR-539-5p-CENPF axis regulates hypoxia-induced stemness in LUAD.	8 (4/2/2)
Hui et al.^36^	Retrospective, TCGA/GEO-based	NR (TCGA training and GEO validation)	LUAD	4	RNA-seq	Signature distinguishes OS (p < 0.0001 TCGA, p = 0.0032 GEO); drug sensitivity differences.	Three hypolncRNA-regulated subtypes; 4-hypolncRNA signature for prognosis and personalized therapy in LUAD.	9 (4/2/3)
Zhu et al.^37^	Retrospective, TCGA/GEO-based	386 (193 training and 193 validation)	LUAD	5	RNA-seq	Pyroptosis-related signature: linked to OS and immunotherapy response.	Pyroptosis-related signature predicts OS and immunotherapy in LUAD; differences in TME between subtypes.	8 (4/2/2)

lncRNAs: long noncoding RNAs; LUAD: lung adenocarcinoma; LUSC: lung squamous cell carcinoma; TCGA: The Cancer Genome Atlas; GEO: Gene Expression Omnibus; GTEx: Genotype-Tissue Expression; NOS: Newcastle-Ottawa Scale; GSEA: Gene Set Enrichment Analysis; OS: overall survival; HR: hazard ratio; TME: tumor microenvironment; PR: pyroptosis-related; PCD: programmed cell death; IH: intermittent hypoxia; ceRNA: competing endogenous RNA; RNA-seq: RNA sequencing; KM: Kaplan-Meier; TMB: tumor mutational burden; TIDE: Tumor Immune Dysfunction and Exclusion; and NR: not reported. *All of the clinical cohort studies included in the present study were conducted in China, the exception being the study by Wang et al.,^31^ which included cohorts from China and the UK. All 18 studies had a retrospective design; dataset labels (TCGA and GEO) indicate the public transcriptomic cohorts used for model derivation and/or validation.

**Chart 2:** Narrative mapping of eligible experimental studies evaluating long noncoding RNA-mediated regulation of hypoxia-related or pyroptosis-related pathways in lung cancer.

Study*	Study Design	Histological Subtype	Cell Lines	No. of lncRNAs	Expression Method	Hypoxia Evidence	Pyroptosis Evidence	Key Findings and Discussion
Wang et al.^38^	Experimental (in vitro)	NSCLC	A549, SPCA-1	1 (PVT1)	quantitative PCR, Western blot	Hypoxia upregulates PVT1.	NA (focus on HIF-1α expression, not pyroptosis).	PVT1 promotes HIF-1α expression via ceRNA for miR-199a-5p under hypoxia.
Liu et al.^39^	Experimental (in vitro)	NSCLC	A549, H1975, PC9, SPC-A1, H1299	1 (XIST)	quantitative PCR, Western blot	NA	Downregulated XIST promotes pyroptosis via miR-335/SOD2/ROS pathway.	Knockdown of XIST inhibits NSCLC via miR-335/SOD2/ROS-mediated pyroptosis.
Hua et al.^40^	Experimental (in vitro/in vivo)	NSCLC	A549, H1299, H1650, H1975, PC9	1 (AC020978)	quantitative PCR, Western blot	Hypoxia upregulates AC020978 via HIF-1α.	NA (focus on glycolysis/proliferation, not pyroptosis).	AC020978 promotes proliferation/glycolysis via PKM2/HIF-1α axis; potential therapeutic target for NSCLC.
Li et al.^41^	Experimental (in vitro)	NSCLC	A549, H1650	1 (H19)	quantitative PCR, Western blot	Hypoxia (CoCl_2_) upregulates H19, HIF1-α, HO-1, MRP1, P-gp.	NA (focus on drug resistance/apoptosis, not pyroptosis).	Hypoxia induces H19 and chemoresistance in NSCLC cells; promotes invasion/migration and reduces apoptosis.
Dai et al.^42^	Experimental (in vitro/in vivo)	LUAD/NSCLC	PC9, PC9/GR, H1975, H1975/OR, A549, H1299	1 (LINC00969)	quantitative PCR, Western blot	NA	LINC00969 suppresses NLRP3 expression, inhibiting pyroptosis; promotes gefitinib resistance.	LINC00969 epigenetically represses NLRP3 via EZH2/METTL3, inhibiting pyroptosis and promoting TKI resistance; potential biomarker/therapeutic target.
Yang et al.^43^	Experimental (in vitro/in vivo)	NSCLC/LUAD	A549, H460, H1299, H1975, PC9, SPC-A1	1 (HIF1A-As2)	quantitative PCR, Western blot	KRAS-responsive; high in lung cancer.	NA (focus on proliferation/EMT, not pyroptosis).	HIF1A-As2/MYC double-loop promotes proliferation/metastasis via DHX9; sensitizes to inhibitors.
Chu et al.^44^	Experimental (in vitro)	NSCLC	A549, THP-1	4 (NDRG1 transcripts)	RNA sequencing, quantitative PCR	Hypoxia induces NDRG1 lncRNAs.	NA (focus on EVs/progression, not pyroptosis).	M2 macrophage-derived EVs promote NSCLC progression via NDRG1-009-miR-34c-5p-VEGFA etc. under hypoxia.
Zhang et al.^45^	Experimental (in vitro/in vivo)	LUAD	PC9, PC9/OR, H1975, H1975/OR, HCC827, HCC827/OR	1 (APCDD1L-AS1)	quantitative PCR, Western blot	Hypoxia-inducible via HIF-1α.	NA (focus on TCA cycle/osimertinib resistance, not pyroptosis).	HIF-1α/APCDD1L-AS1/DLST axis promotes TCA cycle/osimertinib resistance; potential therapeutic target.
Liu et al.^46^	Experimental (in vitro/in vivo)	NSCLC	A549, A549/CDDP, H460, H460/CDDP	1 (lncRNA16)	quantitative PCR, Western blot	NA	lncRNA16 inhibits pyroptosis via microRNA1827/MBD3/GSDME.	lncRNA16 promotes platinum resistance by inhibiting pyroptosis; microRNA1827 agomir sensitizes.

lncRNAs: long noncoding RNAs; NSCLC: non-small cell lung cancer; LUAD: lung adenocarcinoma; CoCl_2_: cobalt chloride; GR: gefitinib-resistant; OR: osimertinib-resistant; CDDP: cisplatin-resistant; EV: extracellular vesicle; TCA: tricarboxylic acid; NA: not applicable (pathway not assessed); HIF-1α: hypoxia-inducible factor 1-alpha; ceRNA: competing endogenous RNA; SOD2: superoxide dismutase 2; ROS: reactive oxygen species; PKM2: pyruvate kinase M2; HO-1: heme oxygenase-1; MRP1: multidrug resistance-associated protein 1; P-gp: P-glycoprotein; EZH2: enhancer of zeste homolog 2; METTL3: methyltransferase-like 3; TKI: tyrosine kinase inhibitor; EMT: epithelial-mesenchymal transition; DHX9: DExH-box helicase 9; NDRG1: N-myc downstream regulated gene 1; VEGFA: vascular endothelial growth factor A; DLST: dihydrolipoamide S-succinyltransferase; MBD3: methyl-CpG binding domain protein 3; GSDME: gasdermin E; and PH: pulmonary hypertension. Note: Chart 2 includes direct integrative studies and indirect mechanistic studies. NA indicates that the corresponding pathway was not assessed in that study; such studies were retained to map mechanistic evidence relevant to the proposed hypoxia-pyroptosis crosstalk framework rather than to imply direct within-study demonstration of both processes. *All studies were conducted in China, the exception being the study by Yang et al.,^43^ which was performed in China, the UK, and Italy.

### OS analysis

The pooled prognostic value of HP-lncRNA signatures for OS in lung cancer was synthesized from 14 studies. The random-effects model of crude HRs indicated that high expression of HP-lncRNAs was significantly associated with poorer OS (pooled HR = 1.53; 95% CI, 1.24-1.90; p < 0.001; Figure S1). This association remained statistically significant when we analyzed HRs that were adjusted for key clinicopathological covariates in multivariate analyses (pooled adjusted HR = 1.50; 95% CI, 1.23-1.84; p < 0.001; Figure 2). Heterogeneity was substantial in both crude and adjusted random-effects models (I^2^ > 99%; p < 0.001). Accordingly, the pooled estimate should be interpreted with caution because it reflects a broad summary across biologically and methodologically heterogeneous HP-lncRNA signatures rather than a single standardized prognostic biomarker. 

**Figure 2:**
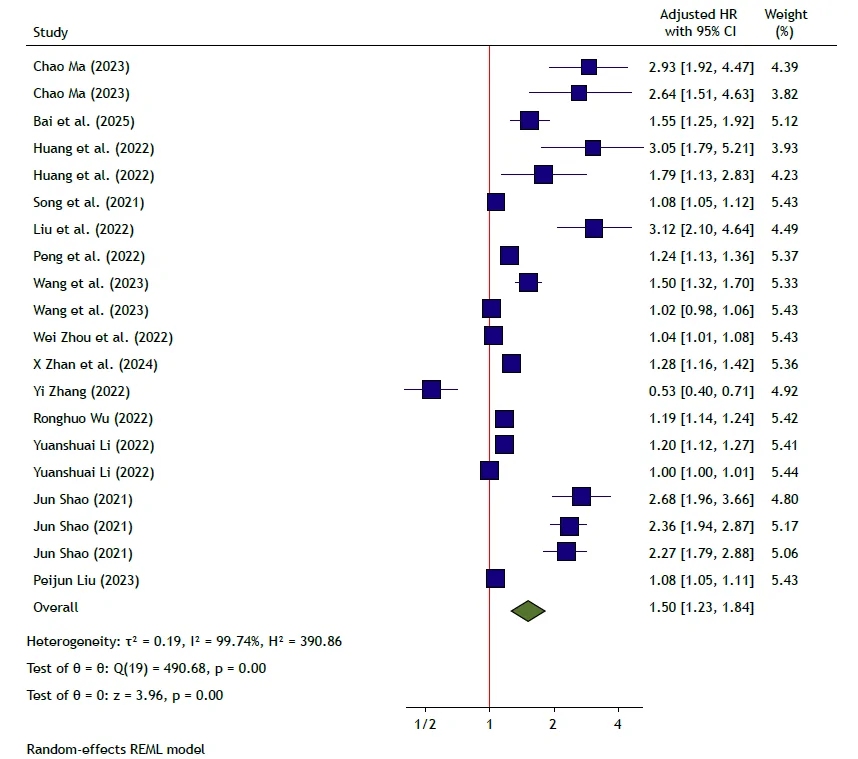
Forest plot for the association between high-risk hypoxia-pyroptosis-associated long noncoding RNA signatures and overall survival in lung cancer: multivariate-adjusted analysis. Hazard ratios (HRs) and 95% CIs are derived from multivariate Cox regression models reported in each included study.

### Subgroup analysis

In subgroup analysis by histological type (Figure 3), the random-effects model showed a significant prognostic effect in LUAD for both crude (HR = 1.68; 95% CI, 1.37-2.06; p < 0.001) and adjusted estimates (adjusted HR = 1.64; 95% CI, 1.34-1.99; p < 0.001). In contrast, the pooled effect for LUSC was not statistically significant (crude HR = 0.88; 95% CI, 0.51-1.53; adjusted HR = 0.91; 95% CI, 0.55-1.50). The test for subgroup differences was statistically significant (Q = 4.63, p = 0.03 for crude; and Q = 4.58, p = 0.03 for adjusted), indicating that the prognostic strength of HP-lncRNA signatures differs significantly between LUAD and LUSC subtypes, with a more robust adverse association observed in LUAD. 

**Figure 3:**
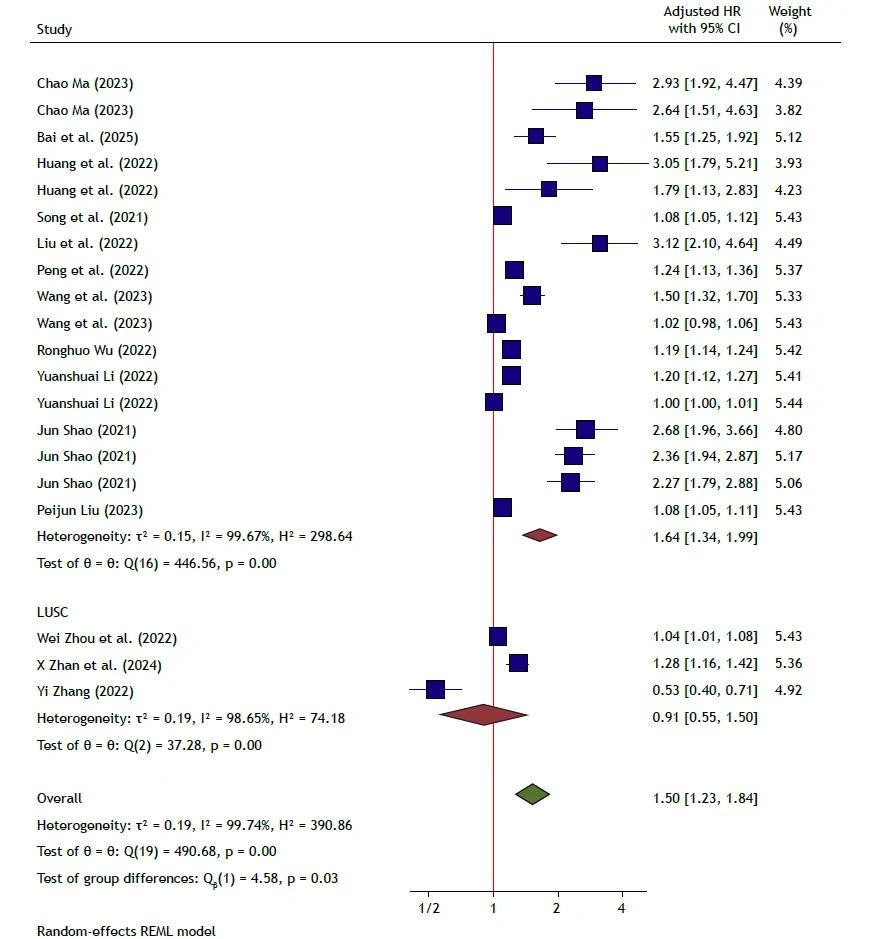
Subgroup meta-analysis of the prognostic value of hypoxia-pyroptosis-associated long noncoding RNA signatures for overall survival, stratified by histological subtype: multivariate-adjusted hazard ratios (HRs). LUAD: lung adenocarcinoma; and LUSC: lung squamous cell carcinoma.

With regard to study design, subgroup analysis of the different prognostic cohorts (training, validation, or combined) revealed consistently significant adverse associations in the training cohorts (adjusted HR = 2.03; 95% CI, 1.37-2.99) and validation cohorts (adjusted HR = 1.58; 95% CI, 1.04-2.35). However, the pooled effect for studies that did not separate cohorts was nonsignificant (adjusted HR = 1.28; 95% CI, 0.98-1.68; Figure S2A). Similar trends were observed in the analysis of crude HRs (Figure S2B). The test for subgroup differences was not statistically significant (Q = 3.82, p = 0.16 for adjusted; and Q = 3.88, p = 0.14 for crude), suggesting that the prognostic performance of the signatures is consistent across different analytical cohort designs. 

Exploratory stage-stratified analysis suggested that elevated expression of HP-lncRNA signatures was associated with poorer OS in both early-stage (I-II) and advanced-stage (III-IV) disease. The point estimate appeared numerically larger in the advanced-stage subgroup; however, the direction of effect was similar across stage categories, and no clear evidence of stage-specific effect modification was observed. In the advanced-stage subgroup (III-IV), elevated HP-lncRNA expression remained significantly associated with poorer OS (adjusted HR = 1.60; 95% CI, 1.44-1.77; p < 0.001; Figure S3). Moderate heterogeneity was observed among the study estimates (I^2^ = 59.72%). Although these findings suggest persistence of the adverse prognostic association in advanced-stage disease, they should not be interpreted as definitive evidence that stage modifies the prognostic effect. 

### Sensitivity analysis

A leave-one-out sensitivity analysis was performed to assess the robustness of the primary pooled estimates. Sequentially omitting each individual study from the meta-analysis did not substantially alter the results. The recalculated pooled HRs for both crude and adjusted analyses remained statistically significant (p < 0.001 for all) and within a narrow range (crude HR: 1.48-1.61; adjusted HR: 1.45-1.57; Figure S4A-B). These findings indicate that the observed adverse association between high-risk HP-lncRNA signatures and OS was not driven by any single study. However, this stability should be interpreted alongside the very high between-study heterogeneity, which limits the precision and generalizability of the pooled estimate. 

### Publication bias

Visual inspection of the funnel plot for crude HRs (publication bias crude HR) revealed asymmetry, suggesting the possible presence of publication bias (Figure S5A-B). This was supported by Egger’s linear regression test (p < 0.05). To address this, the trim-and-fill method was applied, which imputed three hypothetical studies to correct the asymmetry. After adjustment, the pooled crude HR remained statistically significant and similar in magnitude (adjusted HR = 1.48; 95% CI, 1.20-1.83). The consistency of the adjusted HR before and after imputation indicates that, although some publication bias may be present, it does not substantially alter the overall conclusion of a significant association between high-risk HP-lncRNA signatures and poorer OS in lung cancer. 

### Systematic review of experimental studies

We included and evaluated nine experimental studies in our qualitative synthesis. Those studies provided mechanistic evidence relevant to hypoxia-associated lncRNA regulation, pyroptosis-associated lncRNA regulation, and their possible biological convergence in lung cancer models, primarily NSCLC and LUAD (Chart 2). 

Several studies supported a hypoxia-related role for specific lncRNAs in lung cancer. Hypoxia, induced by cobalt chloride exposure or low-oxygen culture conditions, upregulated multiple oncogenic lncRNAs, including *PVT1*, *AC020978*, *H19*, and *APCDD1L-AS1*. These lncRNAs promoted tumor adaptation through distinct mechanisms, such as stabilization of HIF-1α signaling via competing endogenous RNA activity (*PVT1*); enhancement of glycolysis and proliferative capacity through the *AC020978*-PKM2/HIF-1α axis; induction of invasion, migration, and cisplatin resistance in *H19*-overexpressing cells; and reprogramming of the tricarboxylic acid cycle leading to osimertinib resistance through *APCDD1L-AS1*-DLST stabilization. In addition, HIF-1α-AS2 was shown to form a KRAS-responsive double-positive feedback loop with MYC, thereby promoting proliferation and epithelial-mesenchymal transition, while hypoxia-responsive NDRG1-related lncRNA transcripts were implicated in microenvironmental remodeling through M2 macrophage-derived extracellular vesicle signaling and downstream VEGF-A/EGLN3-related pathways. Although these findings support a broad role for lncRNAs in hypoxic adaptation and tumor progression, they do not by themselves establish pyroptosis regulation.^38^ ^,^ ^40^ ^,^ ^41^ ^,^ ^43^ ^-^ ^45^

Other studies supported a pyroptosis-related role for specific lncRNAs in lung cancer. In particular, *XIST* was reported to inhibit pyroptotic cell death through the miR-335/SOD2/ROS pathway; *LINC00969* epigenetically repressed *NLRP3* through EZH2/METTL3-dependent mechanisms to inhibit pyroptosis and promote gefitinib resistance, and *lncRNA16* suppressed the MBD3/GSDME axis by sponging miRNA1827, thereby promoting platinum resistance. These studies provide direct evidence that certain lncRNAs can suppress pyroptosis-associated signaling in lung cancer, although they were not conducted as integrated hypoxia-pyroptosis models.^39^ ^,^ ^42^ ^,^ ^46^

Taken together, the nine included studies suggest potential convergence between hypoxia-related lncRNA dysregulation and pyroptosis-associated signaling, but direct experimental evidence remains limited. Most studies examined one side of this proposed crosstalk in isolation: hypoxia-related induction and tumor adaptation on the one hand, or pyroptosis suppression and treatment resistance on the other. Although targeted interventions such as *APCDD1L-AS1* knockdown, *LINC00969* silencing, or restoration of miRNA1827 activity in lncRNA16-driven models partially reversed malignant phenotypes and, in some settings, restored pyroptosis-related signaling, the current evidence is best interpreted as a narrative mechanistic mapping rather than uniform proof of a fully established hypoxia-lncRNA-pyroptosis axis in lung cancer.^42^ ^,^ ^45^ ^,^ ^46^

### Integrated functional features of HP-lncRNAs in studies

Table S1 provides an integrative summary of lncRNAs (n = 113) that are associated with both pyroptosis and hypoxia in LUAD. For each lncRNA, literature support, tumor versus normal expression, and functional roles in prognostic signatures are summarized. In addition, the correlation profiles between the lncRNA and its related genes were systematically summarized, and the pyroptosis- and hypoxia-related highly associated genes were highlighted. The dominant biological axis was inferred on the basis of these correlation patterns for each lncRNA, which allowed the classification into hypoxia-dominant, pyroptosis-dominant, or hypoxia-pyroptosis crosstalk categories. This integrative framework helps to interpret more clearly how single lncRNAs can together control the inflammatory cell death and hypoxic stress responses in LUAD. 

### Protein-protein interaction network revealing hypoxia-pyroptosis crosstalk modules

A protein-protein interaction network was constructed by using genes that correlated strongly with the prognostic lncRNAs. Analysis revealed distinct functional modules: a hypoxia/HIF-1 and glycolysis cluster (centered on HIF-1α , EPAS1, HK2, and LDHA); a dense inflammasome-pyroptosis module (NLRP family, caspase-1/4/5, PYCARD, IL1-α/β, and IL-18); and modules for gasdermins and mitochondrial stress-autophagy (GSDMA/B/C/E and BNIP3/BNIP3L). HIF-1α and caspase-1 emerged as central hubs, directly linking hypoxic metabolic reprogramming to inflammasome activation and providing a mechanistic bridge for lncRNA-mediated hypoxia-pyroptosis coordination (Figure 4). 

**Figure 4:**
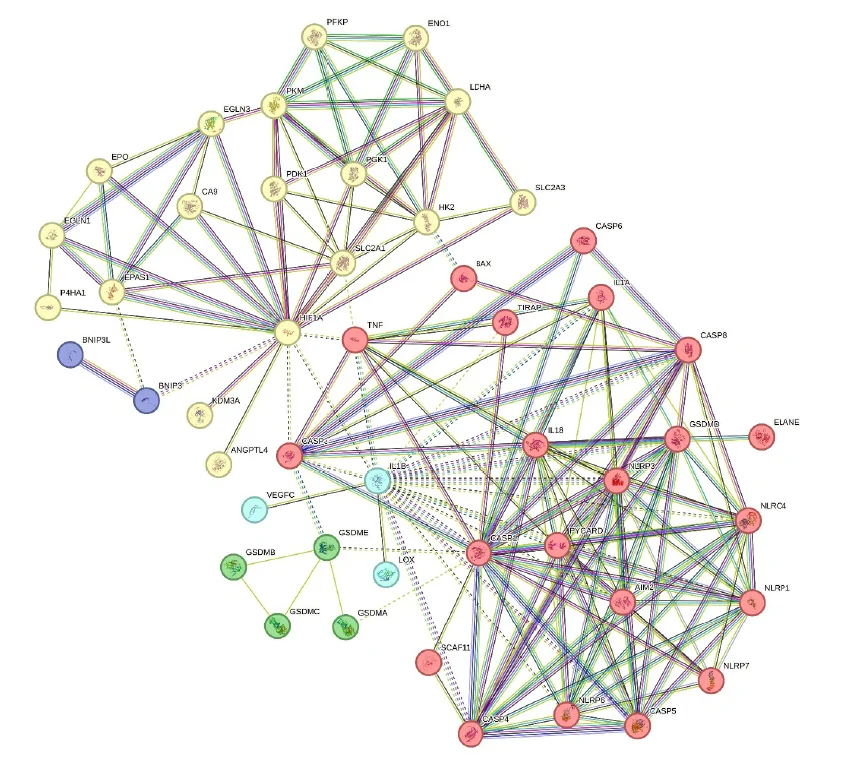
Search Tool for the Retrieval of Interacting Genes/Proteins (STRING)-derived protein-protein interaction network reveals structured crosstalk between hypoxia and pyroptosis signaling pathways. The Markov Cluster Algorithm was used in order to cluster the network, which resulted in the identification of different functional modules such as HIF-1 signaling/glycolysis, inflammasome-mediated pyroptosis, gasdermin family, and mitochondrial stress-autophagy pathways. The size of the nodes indicates the centrality of the network and brings into light the important hub proteins that connect hypoxic adaptation to the processes of inflammatory cell death.

### Functional enrichment analysis highlighting hypoxia-pyroptosis convergence

Functional enrichment analysis of proteins correlated with the prognostic lncRNAs revealed significant convergence on hypoxia and pyroptosis pathways. Reactome pathway analysis demonstrated strong enrichment for pyroptosis, regulated necrosis, inflammasome signaling, IL-1 processing, and HIF-mediated transcriptional regulation (Figure S6). This coenrichment suggests that inflammatory cell death programs are closely coupled with metabolic adaptation to hypoxic stress. Gene Ontology cellular component analysis further highlighted the central role of inflammasome structures, with significant enrichment for the inflammasome complex and its specific subtypes (NLRP3, AIM2, NLRP1, and IPAF), as well as caspase-containing complexes (Figure S7). Enrichment of cytosolic and organelle outer membrane terms also supports the importance of cytoplasmic and mitochondrial platforms in executing these pathways. Consistent with this, Gene Ontology biological process terms were predominantly related to pyroptosis, positive regulation of IL-1β production, inflammasome assembly, and cysteine-type endopeptidase activity. Notably, “cellular response to hypoxia” was also significantly enriched (Figure S8), corroborating the functional integration of hypoxic stress responses with inflammasome activation. Collectively, these enrichment patterns provide consistent evidence that hypoxia-driven metabolic reprogramming and inflammasome-mediated pyroptosis are coregulated within the protein networks associated with the prognostic lncRNAs. 

## DISCUSSION

Our findings in this comprehensive systematic review and meta-analysis establish HP-lncRNAs as significant prognostic biomarkers and mechanistic regulators in lung cancer. Our quantitative synthesis demonstrated that high HP-lncRNA expression is independently associated with 50% poorer OS, particularly in patients with LUAD (adjusted HR = 1.64) and those with disease that is more advanced (stages III-IV; adjusted HR = 1.60). Qualitatively, we identified convergent but mostly indirect mechanistic evidence suggesting that hypoxia-responsive lncRNAs and pyroptosis-regulating lncRNAs may participate in a shared biological crosstalk network; however, direct within-study confirmation of a unified hypoxia-lncRNA-pyroptosis axis remains limited. Integrated bioinformatics revealed HIF-1α and caspase-1 as central hubs, linking hypoxic adaptation to inflammasome activation, thereby providing a unified molecular framework for HP-lncRNA function in tumor progression and therapeutic resistance. 

The pooled HRs from our meta-analysis (adjusted HR = 1.50; 95% CI, 1.23-1.84) indicate that high-risk HP-lncRNA expression profiles are strongly associated with worse OS in lung cancer patients, supporting their clinical utility as independent prognostic biomarkers for risk stratification beyond traditional clinicopathological factors. This effect size is consistent with previous meta-analyses^47^ demonstrating that dysregulated lncRNA expression is generally associated with adverse survival outcomes in NSCLC, emphasizing the prognostic significance of lncRNA-based signatures. These findings suggest that expression of high-risk signatures lncRNA could identify patients with more aggressive disease who may benefit from intensified surveillance or adjuvant therapies.^3^

The results of our subgroup analysis show that the prognostic association was subtype-specific, with a significant adverse association in LUAD but not in LUSC. This difference is likely due to the fundamental biological and molecular heterogeneity between these major NSCLC subtypes.^48^ ^,^ ^49^ LUAD is characterized by distinct driver mutations (e.g., EGFR and KRAS) and a TME that may be more responsive to hypoxic stress and inflammatory pathway modulation, whereas LUSC may rely on alternative oncogenic pathways where the hypoxia-pyroptosis axis is less dominant.^50^ Moreover, the differential lncRNA expression patterns and subtype-specific oncogenic drivers documented in previous The Cancer Genome Atlas-based analyses underscore the need for histology-stratified biomarker development.^51^

Exploratory stage-stratified analysis suggested that the adverse prognostic association remained evident in advanced-stage disease (III-IV; adjusted HR = 1.60). However, because no clear evidence of stage-specific effect modification was observed, this finding should be interpreted cautiously as subgroup persistence rather than proof that stage alters the prognostic effect of HP-lncRNA signatures. This pattern is nonetheless consistent with the broader role of hypoxia in tumor progression, metastasis, and therapy resistance in advanced cancers.^3^ ^,^ ^52^ ^-^ ^54^

The robustness of our overall analysis is supported by several factors. Sensitivity analysis confirmed that no single study excessively influenced the pooled estimate. Subgroup analyses demonstrated consistent adverse associations across different analytical cohort designs (training vs. validation). Although funnel plot asymmetry suggested potential publication bias, application of the trim-and-fill method yielded an adjusted HR (= 1.48) that remained statistically significant and materially unchanged, indicating that the core finding is valid despite this limitation.^19^

The prognostic significance of HP-lncRNAs is supported by convergent experimental observations linking hypoxic stress, lncRNA dysregulation, and avoidance of inflammatory cell death, although a fully integrated experimentally validated framework remains incomplete. Hypoxia, primarily through the stabilization of HIF-1α, serves as a strong transcriptional inducer for various lncRNAs.^53^ ^,^ ^54^ Our review confirms that lncRNAs such as *PVT1* and *APCDD1L-AS1* are directly upregulated by HIF-1α binding, playing critical oncogenic roles.^38^ ^,^ ^45^ These hypoxia-induced lncRNAs promote tumor adaptation by enhancing glycolytic metabolism (e.g., the *AC020978*-PKM2 axis), facilitating proliferation, and creating feedback loops that maintain stemness and epithelial-mesenchymal transition, thereby driving metastasis and contributing to resistance against therapies like osimertinib and cisplatin.^40^ ^,^ ^41^ ^,^ ^43^ Importantly, a major way these lncRNAs help tumors survive stress is through the active inhibition of pyroptosis. Experimental studies consistently show that oncogenic lncRNAs act as molecular repressors of the pyroptotic pathway. For example, *LINC00969* silences the *NLRP3* inflammasome gene epigenetically via EZH2/METTL3-mediated histone and RNA methylation,^42^ while lncRNA16 sequesters miRNA1827 to downregulate the essential pyroptosis executor GSDME.^46^ Similarly, knocking down *XIST* enhances pyroptotic cell death through the miR-335/SOD2/ROS pathway.^39^ This cumulative evidence positions lncRNAs as critical players in dampening the caspase-1/GSDMD-dependent lytic cell death pathway, enabling tumor cells to survive in hostile microenvironments.^7^ ^,^ ^55^ This clarified mechanism highlights a promising therapeutic vulnerability. The association between HP-lncRNA overexpression and therapy resistance, along with evidence that their targeted inhibition (e.g., knocking down *APCDD1L-AS1* or silencing *LINC00969*) can resensitize tumor cells and partially restore pyroptotic signaling, suggests a strategic approach to overcoming treatment failure.^42^ ^,^ ^45^ ^,^ ^46^ By pharmacologically disrupting the hypoxia-lncRNA-pyroptosis axis, it might be possible to reactivate this immunogenic cell death pathway. This could not only lead to direct elimination of tumor cells but also enhance antitumor immunity through the release of proinflammatory cytokines, thereby improving the effectiveness of existing chemotherapy and immunotherapy treatments.^6^ ^,^ ^56^ ^,^ ^57^

The prognostic significance of HP-lncRNAs is further contextualized by their potential role in sculpting the TME. The hypoxic TME is a critical orchestrator of immune evasion, and the HP-lncRNA axis identified in the present study is likely to contribute to this immunosuppressive landscape. Systematic, multidimensional assessment of the TME using integrated analytical frameworks, such as immuno-oncology biological research, has demonstrated that immune phenotypes, stromal interactions, and ligand-receptor communication networks are key determinants of immunotherapy response.^58^ Within this context, alterations in inflammatory signaling pathways are particularly informative. For example, mutations in the TNF-α signaling pathway have been associated with enhanced tumor immunogenicity, increased infiltration of effector immune cells (including CD8^+^ T cells and M1 macrophages), and improved survival in NSCLC patients treated with immune checkpoint inhibitors, highlighting the ability of intrinsic tumor signaling to shape an immune permissive microenvironment.^59^ Conversely, suppression of pyroptosis, a form of immunogenic cell death that promotes cytokine release and antigen exposure, represents a potent mechanism of immune escape. Emerging evidence indicates that metabolites within the TME can directly modulate this process; notably, the immunometabolite 4-octyl itaconate inhibits granzyme-A-dependent cleavage of GSDMB, thereby restricting GSDMB-mediated pyroptosis and dampening inflammatory amplification.^60^ These findings suggest a dynamic interplay in which metabolic adaptations associated with hypoxia may further constrain immunogenic cell death. Taken together, our results support a conceptual model in which hypoxia-driven lncRNA expression suppresses multiple pyroptotic pathways, including both inflammasome-associated (e.g., NLRP3/GSDMD) and granzyme-dependent (e.g., GSDMB) mechanisms, while coinciding with broader metabolic reprogramming. This coordinated regulation is consistent with the establishment of an immune-suppressed TME that limits effective inflammatory signaling and reduces sensitivity to immunotherapy. More broadly, accumulating evidence indicates that the efficacy of immunotherapy is critically dependent on the cellular and cytokine composition of the TME, underscoring the need to interpret hypoxia- and pyroptosis-associated biomarkers within a comprehensive immunological framework.^61^ ^,^ ^62^ Therefore, the HP-lncRNA signature may serve as a proxy for a multifaceted immunosuppressive program, one that not only protects tumor cells from lytic death but also actively impedes the function of cytotoxic immune cells.^63^ Therapeutic disruption of this axis could thus work synergistically with immune checkpoint blockade by simultaneously removing a key survival mechanism of cancer cells and alleviating suppression within the immune compartment. 

Beyond HP-lncRNA, an expanding body of evidence indicates that lung cancer prognosis and therapeutic resistance are governed by a broader regulatory network involving protein-coding genes, microRNAs, and microenvironment-responsive therapeutic strategies. For example, recent transcriptomic analyses have identified tetratricopeptide repeat domain 21A (*TTC21A*) as a favorable prognostic biomarker in lung adenocarcinoma, where higher *TTC21A* expression correlates with prolonged survival and increased immune infiltration, including B cells, T cells, neutrophils, and mast cells. This finding underscores the importance of immunomodulatory genes in shaping clinical outcomes and complements lncRNA-based prognostic signatures that capture tumor-immune interactions.^64^ In parallel, post-transcriptional regulation by microRNAs has emerged as a critical determinant of drug sensitivity. Notably, miR-4271 has been shown to enhance the sensitivity of NSCLC to gefitinib by directly targeting the constitutive androstane receptor (CAR), thereby reducing CAR-mediated drug metabolism and clearance. This microRNA-driven mechanism highlights how noncoding RNAs beyond lncRNAs can modulate resistance to targeted therapies through regulation of downstream metabolic pathways.^65^ Furthermore, given that hypoxia is a defining feature of the lung TME, advances in hypoxia-responsive drug-delivery systems provide an additional therapeutic dimension. Hypoxia-activated polymeric micelles and related nanocarriers have been designed to selectively release anticancer agents under low-oxygen conditions, thereby improving intratumoral drug accumulation and overcoming hypoxia-associated resistance. Such nanodelivery platforms illustrate how microenvironment-adapted therapeutic strategies can synergize with molecular targeting approaches to enhance treatment efficacy in hypoxic tumors.^66^ These complementary mechanisms reinforce the concept that effective prognostic assessment and therapeutic intervention in lung cancer will likely require integrated consideration of coding genes, diverse classes of noncoding RNAs, and microenvironment-responsive treatment technologies. 

Our integrative network analysis offers a comprehensive view that merges clinical and mechanistic findings. The identification of HIF-1α and caspase-1as central, highly connected hubs within the protein-protein interaction network offers a compelling molecular explanation for the observed hypoxia-pyroptosis crosstalk. HIF-1α, the master regulator of hypoxic adaptation, directly interfaces with proteins involved in glycolytic metabolism (e.g., HK2 and LDHA) and inflammatory signaling.^53^ ^,^ ^54^ Caspase-1, the key effector protease for canonical pyroptosis, sits at the nexus of the inflammasome complex (NLRP3 and PYCARD) and cytokine maturation (IL-1β and IL-18).^7^ ^,^ ^55^ Their central positioning suggests that these hubs act as critical relay points, translating the metabolic stress signal of hypoxia into the regulation of the inflammasome apparatus, thereby coordinating cell fate decisions. Functional enrichment analyses strongly support this integrated model. The significant coenrichment of Gene Ontology terms and Reactome pathways related to both “cellular response to hypoxia” and “inflammasome complex assembly” or “pyroptosis” indicates that these processes are not merely coincidental but are coregulated within the molecular networks linked to prognostic HP-lncRNAs.^55^ ^,^ ^67^ This convergence implies that the adaptation of a tumor cell to low oxygen is mechanistically connected to its ability to suppress an immunogenic form of cell death, a survival strategy reflected by our HP-lncRNA signatures. These bioinformatic insights carry direct translational implications. First, they suggest that HP-lncRNA signatures may capture elements of a broader pathway-level state linking hypoxic adaptation and pyroptosis-related signaling. However, because the prognostic meta-analysis pooled highly heterogeneous multigene signatures, these findings should not be interpreted as validation of a single unified prognostic tool. Second, the elucidated network highlights novel combinatorial therapeutic targets. For example, targeting the HIF-1α pathway (e.g., with HIF inhibitors) alongside pro-pyroptotic agents (e.g., NLRP3 agonists) or specific hub-associated lncRNAs (e.g., utilizing antisense oligonucleotides) could synergistically exploit this axis to induce robust, immunogenic tumor cell death and address the significant resistance typical of hypoxic tumor environments.^6^ ^,^ ^56^ ^,^ ^68^

Translating our findings from research to clinical practice offers significant potential for enhancing lung cancer management. The observed prognostic associations suggest that HP-lncRNA signatures may have potential as noninvasive biomarker candidates. Many lncRNAs are detectable and stable in bodily fluids, making them suitable for liquid biopsy platforms using circulating tumor cells or exosomes. This approach allows dynamic risk assessment, monitoring of minimal residual disease, and early detection of therapeutic resistance, with less patient burden when compared with repeated tissue biopsies.^10^ ^,^ ^69^ Therapeutically, the HP-lncRNA axis allows for a dual targeting strategy. Direct inhibition of oncogenic HP-lncRNAs, such as *APCDD1L-AS1* and *LINC00969*, is increasingly viable through antisense oligonucleotides and clustered regularly interspaced short palindromic repeats (CRISPR)-based silencing technologies (e.g., CRISPR interference), potentially reversing their pro-survival effects.^12^ ^,^ ^68^ Additionally, agents aimed at restoring pyroptosis, such as small-molecule NLRP3 agonists or compounds that enhance GSDMD cleavage, could overcome lncRNA-mediated suppression and activate this immunogenic cell death pathway directly.^7^ ^,^ ^67^ A combinatorial strategy targeting both the upstream lncRNA and the downstream pyroptosis pathway may yield synergistic effects, particularly in hypoxic, treatment-resistant tumors.^6^ ^,^ ^57^ Finally, our results highlight the potential for personalized medicine through HP-lncRNA profiling. The strong subtype-specific (LUAD vs. LUSC) and stage-dependent associations suggest that these signatures can identify patient subgroups most reliant on this survival mechanism. This insight could inform tailored treatment decisions; for instance, patients with advanced LUAD and a high-risk HP-lncRNA signature may benefit from clinical trials combining HIF-pathway inhibitors with immunotherapy, while those with low-risk signatures might avoid unnecessary treatment intensification.^48^ ^,^ ^49^ Integrating HP-lncRNA signatures into molecular diagnostics could enhance prognosis and better match patients with emerging therapies targeting the hypoxia-pyroptosis nexus. 

Several limitations of the present study should be noted. First, there was very high heterogeneity among the included prognostic studies, arising from differences in histological subtype, the number and identity of lncRNAs included in each signature, derivation and feature-selection strategies, detection platforms (e.g., RNA sequencing vs. quantitative RT-PCR), validation design, covariate adjustment, and patient cohort characteristics. Although a random-effects model was used in order to account for between-study variability, the pooled HR should be interpreted as a summary across a heterogeneous set of prognostic signatures rather than as evidence for a single reproducible biomarker class. This heterogeneity limits direct biological comparability across studies and underscores the need for standardized, externally validated HP-lncRNA panels in future research. Therefore, our findings support an overall adverse prognostic signal associated with high-risk HP-lncRNA profiles, but they do not establish a universally applicable clinical signature for routine use. In addition, age and sex could not be reliably examined as effect modifiers, because these variables were reported inconsistently across multivariable models across the included studies. For this reason, we removed the corresponding pooled analyses to avoid overinterpretation. Second, a considerable geographical bias is present, given that all clinical cohort studies providing prognostic data were conducted in China. While this may indicate regional research activity, it restricts the generalizability of our findings to other ethnic and demographic groups. External validation across multicenter and multinational cohorts is crucial to confirm the broader prognostic applicability of HP-lncRNA signatures. Third, while our systematic review provides robust associative evidence, a significant mechanistic evidence gap still exists. Most of the experimental studies examined lncRNA roles in pyroptosis or hypoxia in isolation; only a few utilized integrated models (e.g., hypoxic cell systems with pyroptosis readouts) to demonstrate the hypoxia-lncRNA-pyroptosis causal relationship directly. Future functional studies using physiologically relevant hypoxic models are needed to establish this mechanistic link. Finally, the visual and statistical indicators of publication bias (funnel plot asymmetry and Egger’s test) suggest a potential underrepresentation of smaller studies with null results. However, the trim-and-fill adjusted estimate remained significant and consistent in magnitude, implying that while this bias exists, it is unlikely to undermine our primary conclusion regarding the adverse prognostic association. 

In conclusion, high-risk HP-lncRNA signatures were generally associated with poorer OS in lung cancer, particularly in LUAD and advanced-stage disease. However, because the pooled estimate was derived from a highly heterogeneous group of multigene signatures, these findings should be interpreted as evidence of an overall prognostic trend rather than validation of a single reproducible biomarker class. Mechanistic analyses further suggest potential crosstalk between hypoxia-related lncRNA regulation and pyroptosis-associated signaling, although direct integrative evidence remains limited. Overall, these findings support continued investigation of HP-lncRNA-related biology in lung cancer, while future studies should prioritize prospective multicenter validation, signature standardization, and integrated functional models before clinical translation is considered. 
